# Dynamic Balance in Spinal and Bulbar Muscular Atrophy: Relationship between Strength and Performance of Forward Lunge, Step Up and Over, and Step Quick Turn

**DOI:** 10.1155/2021/2540324

**Published:** 2021-10-23

**Authors:** Joseph A. Shrader, Ashwini Sansare, Vincent Shieh, Joshua G. Woolstenhulme, Julie Rekant, Rafael Jiménez-Silva, Galen O. Joe, Angela Kokkinis, Kenneth H. Fischbeck, Christopher Grunseich, Cristiane Zampieri

**Affiliations:** ^1^Rehabilitation Medicine Department, Clinical Center, National Institutes of Health, Bethesda, MD, USA; ^2^Neurogenetics Branch, National Institute of Neurological Disorders and Stroke, NIH, Bethesda, MD, USA

## Abstract

**Introduction:**

Spinal and bulbar muscular atrophy (SBMA) is a neuromuscular disorder that leads to progressive weakness of bulbar and extremity muscles. Dynamic balance during functional tasks has not been reported in people with SBMA.

**Objectives:**

(1) To evaluate the ability to safely complete a forward lunge (FL), step quick turn (SQT), and step up and over (SUO), (2) to determine the presence and severity of dynamic balance impairments by comparing performance to normative data, and (3) to investigate the relationship between lower extremity strength and ability to complete each task.

**Design:**

Cross-sectional analysis. *Participants*. Fifty-three people with SBMA were included in a cross-sectional analysis. Normative datasets provided by the NeuroCom manufacturer and isometric strength literature facilitated patient comparisons. *Outcome Measures*. Force plate-based dynamic balance measures included FL (distance, impact index, contact time, and force impulse), SQT (turn time and turn sway), and SUO (lift up index, movement time, and impact index). Maximal isometric contractions of knee extensors, ankle dorsiflexors, ankle plantar flexors, and hip extensors were measured with fixed frame dynamometry.

**Results:**

The most difficult test, per completion rate, was SUO (52%), followed by FL (57%) and SQT (65%). *t*-tests revealed significant abnormalities in eight of nine balance variables (*p* < 0.05) accompanied by large Cohen′s *D* effect sizes ≥ 0.8. Receiver operating characteristics analysis showed knee extensor (SUO 95% CI =0.78–1.00, SQT 95% CI =0.64-0.92) and ankle plantar flexor strength (SUO 95%CI = 0.75–0.99, SQT 95%CI = 0.64 − 0.92) significantly discriminated the ability to perform SUO and SQT tests with acceptable to excellent areas under the curve.

**Conclusions:**

Considerable dynamic balance abnormalities were observed. Lower extremity strength helps explain low test completion rates. Patients modified task movement patterns, enabling safe task performance. Study results can help direct patient care and future protocol design for people with SBMA.

## 1. Introduction

Spinal and bulbar muscular atrophy (SBMA), also known as Kennedy's disease (KD) [1], is an X-linked neuromuscular disorder, resulting from a mutation in the androgen receptor gene [2]. Recent research indicates the toxic effects of the mutant gene on skeletal muscle as well as motor neurons, contributing to a pathophysiology characteristic of both a motor neuron disorder and myopathy [3]. SBMA primarily affects males, while female carriers of the mutation are usually unaffected clinically [4]. The disease causes slowly progressive weakness of bulbar and extremity muscles with onset in adulthood. Musculoskeletal presentations include muscle cramps, fasciculation, tremors, weakness, and decreased or absent deep tendon reflexes [5].

Major muscle group weakness leads to considerable limitations of function and endurance in people with SBMA [6]. Falls were reported in 64% of individuals from the placebo group in a recent randomized clinical trial [7]. A study including 223 people with SBMA reported the median age of onset for handrail use on stairs to be forty-nine years, for the use of a cane to be fifty years, and for requiring a wheelchair for primary mobility to be sixty-one years [8]. Common endurance limitations in people with SBMA include difficulty with sustained and repeated movements such as sit-ups, step-ups, and heel raises [6], and walking endurance limitations have also been reported [5, 9].

Characterizing and quantifying functional limitations associated with SBMA are critical in guiding patient care and research. Some performance-based clinical tools have been studied, such as the Adult Myopathy Assessment Tool (AMAT) [6] and the six-minute walking test [9]. Recently, posturography was used to quantify static postural sway in people with SBMA [10]. However, no SBMA research has explored objective dynamic balance assessment during important tasks such as forward lunging, turning while walking, and stepping.

This research uses objective balance variables from tests of forward lunge (FL), step quick turn (SQT), and step up and over (SUO) to evaluate safe task performance and functional impairment severity in people with SBMA. While multiple different tasks would be needed to cover all the diverse aspects of dynamic balance, we selected these three tasks such that they are challenging yet doable by this clinical population; they are representative of activities of daily living that are most affected in patients with SBMA and are standard tests in the NeuroCom system previously used in multiple other clinical populations. Since one of the main characteristics of SBMA is muscle weakness, we investigated the relationship between lower extremity strength and the ability to perform the dynamic balance tasks.

## 2. Methods

### 2.1. Participants

Fifty-three males diagnosed with SBMA, with a mean age of 51.5 ± 3.5 years, BMI: 26.4 ± 3.4 kg/m^2^, and disease duration: 12 ± 4.2 years participated. The present investigation is a cross-sectional analysis of a larger randomized controlled trial conducted during 2011-2014 in a research hospital on the benefits of functional exercise for people with SBMA [11]. Healthy control subjects were not a part of this trial because the functional exercise group in this study was compared to a group who received stretching only. Inclusion criteria required participants to be ambulatory and able to travel to our hospital. All subjects were men over 18 years of age with genetically confirmed diagnosis of SBMA. The balance data were acquired at the baseline assessment. The protocol was approved by the Institutional Review Board, and informed consent was obtained from all participants. We compared balance scores to a normative dataset provided by the manufacturer, since healthy control participants were not included in the exercise trial. The normative dataset includes four age groups: 20-39, 40-59, 60-69, and 70-79 years old. All subjects in the normative dataset were reported to have no current or past diagnosis or injury affecting balance, be taking no medications affecting the central nervous system or known to affect balance or coordination, and have no symptoms of dizziness or lightheadedness, no symptoms suggestive of vestibular or neurological disorders, no psychological disorders including depression, no history of two or more unexplained falls within the past 6 months, and normal vision with or without glasses.

### 2.2. Outcome Measures

Participants performed the FL, SUO, and SQT bilaterally three times each without upper extremity support (NeuroCom Balance Master; previously Natus Medical Inc., Seattle, WA). Each subject received a detailed test description and a demonstration by the examiner. A physical therapist provided safe guarding against falls. Lower extremity strength was tested bilaterally, using a Quantitative Muscle Assessment (QMA) device consisting of a fixed-frame dynamometer (AEVERL Medical, LLC, Gainesville, GA) and load cells (Interface, Scottsdale, AZ) with computer-assisted data acquisition. Below is a description of each outcome measure.

#### 2.2.1. Forward Lunge

Each subject was asked to step forward and lunge as far and as quickly as possible on one leg, while staying upright at the trunk, and then push backward and return to the starting position. Participants were required to bend their lunging knee to some degree for it to be considered a valid trial. For safety reasons, we could not predetermine a cut point for a minimal degree of lunging knee flexion. The variables measured were (a) distance: length of the forward movement of the COG, expressed as a percentage of participants height; (b) impact index: the maximum vertical force exerted by the lunging leg as it contacts the force plate during landing, expressed as a percentage of body weight; (c) contact time: time lapsed, in seconds, between the lunging leg contacting the force plate and leaving the contact surface; and (d) force impulse: total vertical force generated by the lunging leg during the forward lunging and return to starting position phases of the movement, expressed as a percentage of body weight multiplied by the time, in seconds, that the force was exerted (total work). This test has been reported to have good to excellent reliability [12].

#### 2.2.2. Step Quick Turn

Each subject was asked to take two steps on the force plate, make a quick pivot turn of 180 degrees, and walk back to the starting position as fast as possible. The variables measured were (a) turn time, the time to execute the turn portion of the test and (b) turn sway velocity, the distance travelled by the center of gravity (sway path), in degrees/second, during the turn. This test has been reported to have good to excellent reliability for both measures [13].

#### 2.2.3. Step Up and Over

Each subject was asked to step up onto a four-inch curb/step (step up leg), lift the other leg (swing leg) up and over the curb without touching it, and safely lower the swing leg to land on the force plate. The variables of interest were (a) lift up index: maximum force exerted by the step up leg on the curb, expressed as a percentage of the body weight; (b) movement time: time elapsed between the initial weight shift to the nonstepping/swing leg and the impact of the swing leg down onto the force plate; and (c) impact index: the vertical force exerted by the swing leg as it landed on the force plate, expressed as a percentage of the body weight. This test has been reported to have good to very good reliability in healthy adults [12].

#### 2.2.4. Isometric Strength

The average of two maximal voluntary isometric contractions (MVICs) were measured and recorded bilaterally for the following muscle groups: knee extension, ankle dorsiflexion, ankle plantarflexion, and hip extension [11]. Additionally, these four muscle groups were summed bilaterally to create a lower extremity strength composite score for each subject. Individualized strength prediction values were derived for each muscle considering gender, height, weight, age, and body side using published normative dataset equations [14]. A ratio (percent of predicted MVIC) was then calculated for individual muscle groups (right and left) and the composite value (averaged right and left) for each subject.

### 2.3. Statistical Analysis

Data were dichotomized into able participants who completed all three trials of a test bilaterally, without loss of balance, or not able participants who required reaching for upper extremity support, therapist assistance, or took a corrective step to prevent a fall. Mean differences between people with SBMA who were able to complete each test and the normative database were explored with independent sample *t*-test. Data analysis followed age subgroups determined by the normative database. Differences between people with SBMA able and not able to complete dynamic balance tasks by age, age at disease onset, and disease duration were also explored with an independent sample *t*-test. Analyses were performed with R software (R Foundation for Statistical Computing, version 3.6.0, Vienna, Austria). Cohen's *D* values were calculated to determine the effect size of each measure with the following interpretation criteria: small (0.20), moderate (0.50), and large (0.80) [15]. Additionally, a receiver operating characteristic (ROC) curve analysis was completed to evaluate the discriminatory value of lower extremity strength on ability to complete the functional tests (Epi: a package for statistical analysis in epidemiology, R package version 2.40). We interpreted the data as follows: an area under the curve (AUC) of 0.5 suggests no discrimination (i.e., ability to discriminate patients able and unable to complete the test), 0.5-0.7 is considered poor, 0.7 to 0.8 acceptable, 0.8 to 0.9 excellent, and more than 0.9 outstanding [16]. Sensitivity and specificity were calculated post hoc for tests that demonstrated excellent discriminatory value. We analyzed the right side of the body for all variables, except composite values, because we noticed no asymmetries when strength data was examined.

## 3. Results

The most difficult test for our cohort, based on completion rate, was the SUO (52%), followed by FL (57%) and SQT (65%). Comparisons between people with SBMA able to complete each test and the normative database are shown in Table 1.

Data were compared to two age range groups (40-59 and 60-69 years old). Two participants younger than 40 and one subject older than 69 were excluded. Significant abnormalities were found for nearly all measures. Our cohort lunged forward with significantly shorter COG displacement (distance, *p* < 0.001 for groups 40-59 and 60-69 years old), landed more softly (impact index, *p* < 0.001 for both groups), and spent longer time (contact time, *p* ≤ 0.001 for both groups) in the lunge position. The group with ages 40-59 also performed more work during the forward lunge (force impulse, *p* < 0.001), and a similar finding towards larger force impulse was observed for the older group; however, those results did not reach statistical significance (*p* = 0.06). Large effect sizes (Cohen's *D* > 0.8) were found for all variables of FL and nearly all age groups. Significant abnormalities were also observed for the SUO, with the 40-59 years old group, stepping up onto the step with significantly less force on the step up leg (lift up index, *p* < 0.001), taking longer time to execute the entire test (movement time, *p* < 0.001), and landing more softly with the swing leg (impact index, *p* = 0.004). On the other hand, people with SBMA with ages 60-69 years demonstrated significant differences only on SUO lift up index (*p* = 0.006) when compared to the normative dataset. Effect sizes were large for SUO variables in the 40-59 age range and for the lift up index in the 60-69 age range. Contrary to FL and SUO, the only significant finding on the SQT was that people with SBMA with ages 40-59 took significantly more time to execute the 180-degree pivot turn compared to the normative data (turn time, *p* = 0.009), and a large effect size accompanied that difference.

The role of strength in discriminating our participants' ability to complete each functional test is shown in Table 2. Several muscle groups significantly discriminated between those able or not able to perform the SUO and SQT tests, including the composite lower extremities, knee extensors, and ankle plantar flexors, with the only difference being that strength discriminated better for SUO with excellent AUCs (0.80 < AUC < 0.90), whereas the SQT yielded acceptable AUCs (0.70 < AUC < 0.80). The hip extensors were also discriminatory for the SUO test, with acceptable AUCs. Of note, people with SBMA able to perform the SUO test had lost almost half of their predicted strength in some muscle groups (remaining strength ranging from 36% to 56%) except for the hip extensors, while those not able to perform the SUO test had much less strength ranging from 19% to 35% (Table 2).

Additionally, peak knee extensor strength was approximately double in those able to complete the SUO and SQT tests versus those who were not able. Our comparisons between group demographics showed that participants who were not able to perform the SUO test had a significantly longer disease duration than those who were able (19.3 ± 10.5 vs. 12.4 ± 8.5 years, respectively; *p* = 0.039). Finally, the FL test yielded no significant discriminatory value.

Given the excellent discriminatory value found for most muscle groups during the SUO test, we performed a post hoc analysis to identify cutoff values of those muscle groups at optimal sensitivity and specificity. Findings revealed a strength cutoff at 57% of predicted for the lower extremities (sensitivity 0.88, specificity 0.87), 58% for the right knee extensors (sensitivity 0.84, specificity 0.87), and 50% for the right ankle plantar flexors (sensitivity 0.92, specificity 0.73).

## 4. Discussion

This study is the first, to our knowledge, to report objective dynamic balance measures in an SBMA cohort. Results indicate people with SBMA have great difficulty with FL, SQT, and SUO movements. Significant abnormalities in eight of nine balance variables were observed. Those able to perform the tasks utilized altered movement strategies and required more of their available lower extremity strength for safe task completion, due to loss of reserve strength.

Our results suggest ambulatory people with SBMA experienced a high degree of difficulty performing functional balance tasks as only approximately half of the participants could safely complete the tests. These results are not unexpected given that our cohort had substantial lower extremity muscle weakness and is classified as having moderate functional deficits [6] with mean AMAT scores of 31/45, presented in previous work [11].

### 4.1. Comparison to a Normative Dataset

#### 4.1.1. Forward Lunge

We suggest the FL may have been the most difficult task to perform based on large and very large effect sizes seen for all four variables measured. Only 57% of participants were able to complete the test and did so while moving their center of gravity forward by only 33-41% of expected values. This degree of step distance reduction is about four standard deviations below the normative group mean and is a movement strategy that allows for a shallower FL depth reducing the eccentric and concentric demand of the knee extensors. This finding is also not surprising because the quadriceps, which provide the dominant resistance to knee buckling during FL, were weak in this cohort and are often one of the most affected lower extremity muscle groups in people with SBMA [6]. Participants also landed on the lunging limb with only 36-54% of the expected force, utilized 33-46% more time than expected, and performed 17-33% more work to complete the lunge in comparison to normative data.

The increased time our cohort used to perform FL aligns with expectations for noncopers with ACL injury [17]. Alkjaer et al. found longer FL contact time and decreased quadriceps strength among noncopers. Given the role of the quadriceps in decelerating and accelerating the body during FL, the authors surmised, and we agree that increasing FL time is a compensatory strategy that reduces quadriceps muscle power demands. This longer FL time also increased the work (force impulse) performed by our participants. While each subject was instructed to step as far as possible and return to the starting position without extra steps or loss of balance as quickly as possible within safety limits, most were not able to meet the established test standards.

Surprisingly, strength did not discriminate people with SBMA who were able to perform FL from those who were not able. These results may be partially explained by the movement alterations reviewed above, which allowed our cohort to complete the test with less requirement for quadriceps strength. We speculate that early clinical attention to optimize or preserve quadriceps strength, safely refine and augment the modified FL movement, and become aware of individual FL speed and distance limits may help people with SBMA preserve this important functional ability and may decrease fall risk from an unexpected knee buckling occurrence.

#### 4.1.2. Step Up and Over

The step up and over test was also difficult for our participants with a completion rate of only 52%. In a previous study, the majority of people with SBMA required the use of one handrail to successfully step onto a 7-inch step [6]. However, in the current study, hand support was not allowed and thus a four-inch step was used. In contrast, manufacturer control data was derived with an eight-inch step. Participants generated 32-48% less force than expected, took 38% longer to complete the test (younger cohort), and landed on the swing limb with 33% less force (younger cohort). These results are intuitively consistent with the difficulty of this test, which places high demands on postural stability and lower extremity strength generation. The reduced lift up index indicates poor force generation capacity of the lower limb to raise the body weight onto the step, and longer SUO time may indicate less confidence and muscle power generated during the test. Some of these findings are consistent with other studies in Parkinson's disease [18] and Huntington's disease [19], each reporting significantly reduced lift up index and longer SUO times. However, our cohort generated significantly smaller impact index values but only for the group with ages 40-59 years. The literature is unclear about the role of contralateral quadriceps strength on SUO impact forces. Smaller impact “landing” forces may represent an indirect measure of improved contralateral limb eccentric quadriceps strength and postural stability to lower the body from the step onto the force plate softly [20, 21]. However, our younger cohort, having only 46% of mean predicted peak quadriceps strength, was still able to perform the test, but landed softer than expected. Chmielewski et al. reported that quadriceps strength stood out as the main predictor of SUO impact index (but with weaker quadriceps associated with lower impact index) in participants recovering from ACL reconstruction [21]. The impact index was low initially after surgical repair, when quadriceps strength was impaired and normalized six weeks later when quadriceps strength improved to a level similar to the contralateral unaffected limb. Given the considerable muscle weakness seen in our participants, we suggest quadriceps weakness plays an important role in the reduced impact index seen. Utilization of a four-inch step would also likely reduce the impact index.

#### 4.1.3. Step Quick Turn

While our participants were challenged by SQT, it was the most easily completed test (65% completion). The younger group of people with SBMA took significantly longer (88%) than expected to perform the SQT. Surprisingly, turn sway velocity was the only parameter, of nine studied, not significantly different from normative control data. A previous study in Huntington's disease [19] reported significant differences for both turn time and turn sway velocity. A possible reason for differing results in this study may be in the pattern of movement adopted by our participants. Instead of a pivot turn, our participants used a similar strategy to the “en bloc” turn strategy, which is commonly seen in people with Parkinson's disease [22]. Visual inspection of the center of gravity tracings during turning indicates our participants took two or more (usually multiple) small steps, in order to widen the base of support and help maintain the center of gravity within it. We interpret this pattern of movement as a compensatory strategy to prevent postural instability. This pattern of movement may help explain why turn time was significantly increased and had a large effect size in the younger group while turn sway velocity was not significantly different from normative control data in both groups. In summary, our data demonstrated that the majority of ambulatory people with SBMA could successfully complete a SQT task. However, they use altered movement strategies that incorporate a wider base of support and multiple small steps, turning more slowly. This may help explain why completion rate was the highest for SQT.

### 4.2. Role of Strength in FL, SUO, and SQT Performance

The final goal of the study was to explore the discriminatory value of lower extremity strength in determining the participants' ability to safely complete the three dynamic balance tests. Safe execution of these tests requires strength, as well as adequate joint range of motion and stability, unimpaired cognition, lack of extreme pain, adequate motor control, coordination and vision, and many other factors. Our clinical experience treating people with SBMA informs us that muscle force generation appears to be the primary impairment effect on our cohort's task performance. This cohort, therefore, may provide a more direct avenue to examine strength/function relationships compared with other neuromuscular conditions presenting with additional motor control issues or cognitive impairments. Our data suggest that MVIC differences in a number of lower extremity muscles explain why people with SBMA were either able or not able to perform modified versions of SUO and SQT. While we did see increased strength in those able to complete the FL, ROC analysis did not support the ability to discriminate between those able or not able to perform a FL.

The strength/function relationship is the strongest for the SUO, requiring a fundamental amount of LE strength to safely complete the test, which is supported by larger AUC values for all muscles tested except ankle dorsiflexion. The SUO is particularly demanding on quadriceps function because it requires one to lift their bodyweight up onto a step (concentric), stabilize that limb while swinging the opposite limb over the step, and then lower the body onto the force plate (eccentric) without upper extremity support. Our research group previously identified the quadriceps muscle as one of the most affected lower extremity muscle groups in SBMA at only 40% of expected compared to age-matched control participants [5]. The present cohort has similar quadriceps strength loss, although results from our sensitivity and specificity analysis suggest a functionally important threshold of 58% of predicted strength is necessary to complete the SUO test. This threshold may allow clinicians to help identify which participants may be close to losing or gaining SUO ability and provide a target for strengthening interventions. Finally, the SUO test was the only test where hip extension MVIC played a discriminatory role. However, hip extension MVICs were relatively preserved for all people with SBMA when compared to ankle plantarflexion and knee extension MVICs indicating greater reserve strength at this key muscle group, which may help explain why hip extension strength did not discriminate people who were able or not able to perform FL and SQT.

Despite our participants using a modified SQT (slowing turn and using a similar “en bloc” turn strategy to improve postural stability), this modification did not negate the relevance of strength to perform the test, particularly the quadriceps and ankle plantar flexors which showed acceptable discriminatory value (AUC 0.78). Other researchers have found that these two muscles are key in identifying fallers versus nonfallers among healthy elderly [23]. Given that the data presented here reveal the knee extensors and ankle plantar flexors are the weakest muscles measured in this cohort, we suggest their inclusion in targeted strength training programs for people with SBMA.

### 4.3. Limitations of This Study

The original clinical trial for this study did not enroll male healthy controls; therefore, mean normative data supplied by the manufacturer that included combined male and female values were used in our comparisons. However, it is likely that group differences would have been more robust, had we compared our cohort to men only [24]. Also, when meaningful, the balance variables investigated in this study were normalized to body weight or height, which should partially negate gender differences. Unfortunately, the manufacturer provided dataset did not include any BMI data, and therefore, we were unable to make group comparisons between our patient group and the control group. The manufacturer dataset only included 8-inch step control values for the SUO test. Had we included an age-matched control group utilizing the four-inch step, our group comparisons would have been more accurate, but also may have led to a SUO completion rate too low to analyze. Also, the normative data provided only group means and standard deviations, not individual data, which precluded our ability to use nonparametric statistics in a few instances when variables were not normally distributed. The cutoff values derived from the sensitivity and specificity estimations reflect the current dataset and should be verified against additional datasets to validate their discriminatory value. However, these steps are beyond the scope of this paper. Our sample size was relatively small and was reduced further when analysis required stratification by age and by our decision to require completion of all three trials bilaterally to be included in the analysis. Nevertheless, we believe these limitations do not detract from study conclusions given the robustness of our findings.

## 5. Conclusion

This study provides evidence that people with SBMA having remarkable strength and dynamic balance abnormalities can still complete challenging tasks by using alternative movement strategies. Clinicians and researchers can benefit from awareness of these strategies to help people with SBMA improve or maintain dynamic balance task performance and refine study designs in this population, respectively. However, these strategies cannot overshadow the importance of muscle strength. When strength declines from weak to very weak, altered movement strategies are unable to help complete the SUO and SQT tasks. ROC analyses indicated that quadriceps and plantar flexor muscles played a key role in identifying the ability to perform SUO and SQT tasks. More research is needed to find interventions that help people with SBMA maintain or improve strength and identify strength thresholds needed for functional tasks requiring dynamic balance.

## Figures and Tables

**Table 1:** Comparisons between SBMA cohort and NeuroCom normative references on forward lunge, step quick turn, and step up and over tests. Right-sided data are presented.

Parameter	Age group (years)	SBMA cohort		Normative		*p* value	Cohen's *D*
		*n*	Mean ± SD	*n*	Mean ± SD		
Forward lunge							
Distance (% ht.)	40-59	17	16.5 ± 3.5	47	49.7 ± 8.3	**<0.001**	4.5
	60-69	10	17.2 ± 4.5	26	41.9 ± 6.2	**<0.001**	4.2
Impact index (% wt.)	40-59	17	13.5 ± 5.1	47	38.1 ± 11.6	**<0.001**	2.4
	60-69	10	16.0 ± 6.4	26	29.8 ± 14.6	**<0.001**	1.1
Contact time (sec)	40-59	17	1.6 ± 0.4	47	1.1 ± 0.2	**<0.001**	1.9
	60-69	10	1.6 ± 0.3	26	1.2 ± 0.4	**0.001**	1.1
Force impulse (%wt.) × (sec)	40-59	17	159.7 ± 38.2	47	120.1 ± 22.2	**<0.001**	1.4
	60-69	10	163.8 ± 28.9	26	139.6 ± 41.4	0.06	0.6

Step quick turn							
Turn time (sec)	40-59	20	1.5 ± 1.1	47	0.8 ± 0.5	**0.009**	1.0
	60-69	11	1.1 ± 0.5	26	1.1 ± 0.8	0.93	0
Turn sway (deg/sec)	40-59	20	28.5 ± 13.9	47	23.2 ± 8.8	0.13	0.5
	60-69	11	23.6 ± 7.9	26	24.0 ± 10.2	0.91	0

Step up and over							
Lift up index (% wt.)	40-59	17	22.4 ± 6.9	47	46.9 ± 14.1	**<0.001**	1.9
	60-69	8	26.7 ± 9.3	26	39.6 ± 12	**0.006**	1.1
Movement time (sec)	40-59	17	1.8 ± 0.5	47	1.3 ± 0.3	**<0.001**	1.5
	60-69	8	1.6 ± 0.4	26	1.5 ± 0.4	0.42	0.4
Impact index (% wt.)	40-59	17	30.9 ± 15.9	47	45.9 ± 21	**0.004**	0.8
	60-69	8	40.3 ± 12.8	26	48.2 ± 22.3	0.22	0.4

**Table 2:** Muscle strength as a discriminator between those in the SBMA cohort who were able versus those who were not able to complete each balance test. Strength values are expressed as a percentage of predicted. For clarity, only the right side is presented except for averaged composite strength (lower extremities).

Muscle group	Able to complete (mean ± SD)	*N*	Not able to complete (mean ± SD)	*N*	AUC	95% CI
Forward lunge						
Lower extremities	0.50 ± 0.17	28	0.43 ± 0.15	15	0.62	0.43-0.81
Knee extension	0.39 ± 0.18	28	0.32 ± 0.21	15	0.67	0.48-0.87
Ankle dorsiflexion	0.47 ± 0.20	28	0.33 ± 0.17	15	0.67	0.49-0.85
Ankle plantarflexion	0.31 ± 0.20	28	0.26 ± 0.17	15	0.60	0.41-0.80
Hip extension	1.03 ± 0.43	28	0.93 ± 0.21	15	0.47	0.29-0.65

Step quick turn						
Lower extremities	0.52±0.18	26	0.39±0.11	19	0.75	**0.61–0.90**
Knee extension	0.44±0.20	26	0.26±0.12	19	0.78	**0.64–0.92**
Ankle dorsiflexion	0.47±0.20	26	0.36±0.36	19	0.65	0.49–0.82
Ankle plantarflexion	0.34±0.20	26	0.21±0.21	19	0.78	**0.64**–**0.92**
Hip extension	1.03±0.43	26	0.92±0.26	19	0.45	0.28–0.63

Step up and over						
Lower extremities	0.56 ± 0.15	25	0.35 ± 0.10	15	0.89	**0.79–1.00**
Knee extension	0.46 ± 0.19	25	0.24 ± 0.10	15	0.89	**0.78–1.00**
Ankle dorsiflexion	0.47 ± 0.21	25	0.35 ± 0.15	15	0.66	0.48–0.83
Ankle plantarflexion	0.36 ± 0.21	25	0.19 ± 0.15	15	0.87	**0.75–0.99**
Hip extension	1.10 ± 0.39	25	0.78 ± 0.25	15	0.78	**0.64–0.93**

## Data Availability

The de-identified balance and strength data used to support the findings of this study are available from Dr. Grunseich, christopher.grunseich@cc.nih.gov, for researchers who meet the criteria to access the data.
